# Case report: Nighttime media restriction for pediatric insomnia

**DOI:** 10.3389/frsle.2024.1365784

**Published:** 2024-03-14

**Authors:** Yusuke Arai, Daimei Sasayama, Kazuhiro Suzuki, Jun Watanabe, Yuta Kuraishi, Mika Koido, Shinsuke Washizuka

**Affiliations:** ^1^Department of Psychiatry, Shinshu University School of Medicine, Matsumoto, Japan; ^2^Department of Psychiatry, Kurita Hospital, Nagano, Japan; ^3^Department of Child and Adolescent Developmental Psychiatry, Shinshu University School of Medicine, Matsumoto, Japan; ^4^Department of Community Mental Health, Shinshu University School of Medicine, Matsumoto, Japan

**Keywords:** sleep, screen time, sleep disturbances, child psychiatry, insomnia

## Abstract

This report describes the case of a 13-year-old boy with chronic insomnia and increased daytime sleepiness linked to excessive nocturnal media use. Implementing a restriction on digital device usage after 9 pm led to a significant improvement in the sleep duration with no adverse event. Throughout the 16-week treatment period, the total sleep time of the patient normalized, and the daytime sleepiness problem was resolved. This is an indication that the treatment was effective. This case emphasizes the potential of nighttime screentime restriction in treating pediatric chronic insomnia and highlights the importance of addressing screen time in sleep disorder management.

## Introduction

Since the onset of the coronavirus disease 2019 pandemic, there has been a notable increase in the screen time among pediatric populations (Brzek et al., 2021). Extensive research suggests that screen time may have a negative impact on sleep. A previous study suggested that increased screen time is intricately linked to increased daytime sleepiness and severe insomnia in children (Hisler et al., 2020). Furthermore, a recent pediatric study revealed that media use during late evening hours, specifically from 10 pm to 2 am, is associated with a reduction in the total sleep duration as well as a delay in the bedtime (Lee et al., 2023). This decrease in sleep duration leads to increased sleepiness on subsequent days, perpetuating a cycle of increased screen time and sedentary behavior in early childhood (Magee et al., 2014). This reciprocal relationship underscores the bidirectional nature of nocturnal media use and sleep disturbances in children.

In contrast, recent evidence largely contradicts the idea that limiting screen time affects sleep quality and duration in children and adolescents. A recent randomized controlled trial (RCT) on university students found no immediate improvement in the sleep quality following a 1-week social media restriction (Mahalingham et al., 2023). A pilot cluster-randomized study targeting adolescents showed that while a school-based psychological intervention decreased nighttime electronic media use, it did not increase the sleep duration (Das-Friebel et al., 2019). Another trial revealed that boosting physical activity and reducing sedentary time did not significantly enhance the total sleep time (TST) via reduced screen use (Knebel et al., 2020). Furthermore, a cluster-randomized trial examining the effects of limiting recreational screen use on children's and their families' sleep found no significant differences in the electroencephalogram-based sleep outcomes between the intervention and control groups (Pedersen et al., 2022). These RCTs collectively suggest that screen time limitation has a minimal impact on the sleep quality or duration across a broad demographic, i.e., from children to university students. Moreover, to the best of our knowledge, no studies have shown the effectiveness of limiting screen time as a treatment for chronic insomnia in children.

In this case report, we detail the case of a 13-year-old patient with chronic insomnia who experienced an increase in the total sleep duration and a decrease in daytime sleepiness after just 2 weeks of restricting nocturnal media use. This case underscores the need for further research into screen time restriction as a potential targeted treatment for pediatric and adolescent chronic insomnia.

## Case description

### Patient information

A 13-year-old boy with suspected hypersomnia was referred to a sleep clinic. His daytime sleepiness, which began 3 months earlier, included dozing during meals. He reported adequate sleep duration and regular sleep rhythm, which led the pediatrician to suspect hypersomnia and consult a sleep specialist. Accompanied by his mother, the patient visited our clinic; both consented to the publication of this case report. His sleepiness, peaking around 6 pm, often resulted in him dozing whilst watching television or eating; he scored 8 on the Epworth Sleepiness Scale (ESS) (Takegami et al., 2009). He did not experience cataplexy, hypnagogic hallucinations, or sleep paralysis. He stated that he went to bed at 10 pm, fell asleep within 30 min, and averaged ~450 min of time in bed.

During the psychiatric interview, no apparent symptoms of anxiety, depression, or other significant mental health issues were observed. With no significant developmental or family medical history, his academic performance was excellent, and he was a student council member in elementary school. The patient has a calm and cooperative personality according to the mother. The total score of the Strengths and Difficulties Questionnaire (SDQ) (Matsuishi et al., 2008) for the patient was 4 points. Physical examination revealed normal growth, weight, and throat and jaw morphology. Blood tests, including thyroid function tests, and head computed tomography revealed no abnormalities.

### Diagnostic assessment

Based on the information of the patient, differential diagnoses of chronic insomnia, insufficient sleep syndrome (ISS), idiopathic hypersomnia (IH), and delayed sleep-wake phase disorder (DSWPD) (American Academy of Sleep Medicine, 2014) were considered. For diagnostic assessment, we planned to conduct a sleep diary, actigraphy, polysomnography, and a multiple sleep latency test (MSLT). Additionally, considering the potential underlying neurodevelopmental disorders such as autism spectrum disorder and attention-deficit hyperactivity disorder (ADHD) (American Psychiatric Association, 2013), we administered a questionnaire.

While explaining the diagnostic assessment findings, the boy requested a private conversation with the doctor; hence, the mother was asked to leave the room. The boy revealed that he spent ~120 min each night (from bedtime until late at night) watching videos on his smartphone. His smartphone screen time history revealed an average screen time of 143.5 min per day over the past 4 weeks, primarily spanning between 60 min before bedtime and 2 am. He experienced frequent nocturnal awakenings and watched videos on his smartphone during these times. He reported that while an average of his actual time in bed was 450 min per day over the past 4 weeks, his TST was ~390 min.

Based on this information, we considered chronic insomnia to be the most likely diagnosis. The possibilities of IH and DSWPD were deemed less likely. Given that sufficient TIB has been secured, we considered ISS to be negative. The scores from both the Autism Spectrum Quotient (Wakabayashi et al., 2004) and the ADHD Rating Scale (Pappas, 2006) questionnaires were below the cutoff, allowing us to rule out a secondary sleep disorder associated with neurodevelopmental conditions.

With the consent of the patient, we carefully explained the differential diagnosis and diagnostic methods to patient and his mother and made decisions based on an approach of shared decision-making. To rule out DSWPD, a 2-week actigraphy was performed. Additionally, to rule out IH, we decided to perform polysomnography and a MSLT if daytime sleepiness persisted after therapeutic intervention for the suspected chronic insomnia. Following evaluation and therapeutic intervention, DSWPD and IH were ruled out, leading to a definitive diagnosis of pediatric chronic insomnia.

### Therapeutic intervention

The treatment of chronic insomnia was conducted based on “Guidelines for the proper use and withdrawal of sleeping pills” (Japanese Sleep Society, 2014). The guidelines first recommend sleep hygiene education. During the sleep hygiene education, we taught the patient and his mother about the importance of regular exercise, maintaining a consistent diet, and avoiding engaging in thought-provoking activities while in bed. Considering the history of the patient, we identified nighttime media use as the main causal factor for his chronic insomnia; thus, we emphasized the importance of avoiding nighttime media use. Additionally, we explained the findings of a recent cross-sectional study, which showed that children who use screen time restrictions on their digital devices have shorter screen time compared to those who do not (Arai et al., 2023). This led the patient to introspect on the difficulty of self-managing media use. Consequently, as a therapeutic intervention, we initially set a 2-week period during which the automatic screen time restriction feature of the smartphone was used to limit usage after 9 pm. Considering the pediatric status of the patient and prioritizing safety, we did not use sleeping pills.

### Outcomes

The primary outcomes were the changes in sleep variables measured daily via actigraphy over the 2-week period, the average daily screen time recorded on the smartphone each day throughout the 2-week period, and the ESS scores, all assessed before and after the 2-week treatment period. Additionally, we anticipated potential adverse events associated with the screen time restriction, such as an increase in the irritability of the patient owing to restraint and potential conflicts between parent and child regarding media use. At 4-week post-intervention, we assessed the maintenance of treatment adherence, the persistence of treatment effects, and the presence of adverse events by interviewing the patient. Moreover, at 16-week post-intervention, we interviewed the patient to assess the maintenance of treatment adherence and the effects of the therapeutic intervention, factors and environment related to treatment motivation, and any adverse or unexpected events.

### Results and patient perspective

**Figure 1:**
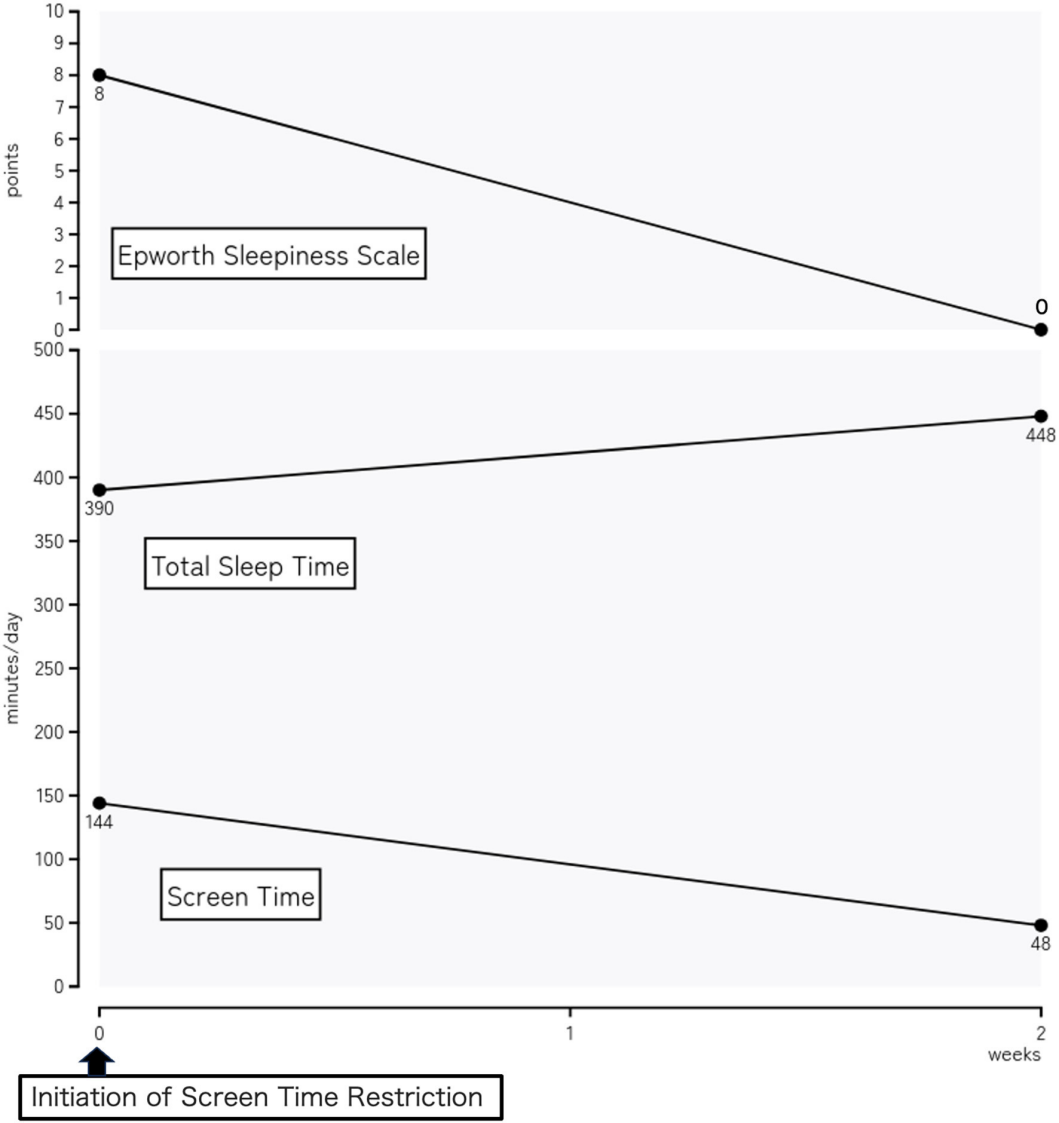
Clinical course of a 13-year-old patient over a 2-week period following the implementation of nighttime media use restrictions. Changes in the sleep patterns (including the total sleep time) and reduction in daytime sleepiness (measured using the Epworth Sleepiness Scale) from the initial consultation to the 2-week follow-up are observed. The daily screen time (minutes) and Epworth Sleepiness Scale score decreased significantly, and the total screen time increased significantly, after the intervention.

Figure 1 shows the clinical course of the patient over 2 weeks after screen time restriction. Within 2 weeks, his sleep pattern normalized (average timing: in bed at 22:18 and out of bed at 5:47, time in bed: 448.71 min, TST: 447.50 min), and daytime sleepiness was not observed (ESS score: 0 points). His screen time was reduced to 47.5 min per day (a decrease of 96.0 min), with no night usage. After confirming the effectiveness of the therapeutic intervention without significant adverse events, we decided to continue its use.

At the 4-week follow-up, he reported a complete resolution of symptoms such as nocturnal awakenings and daytime sleepiness. Furthermore, he added, “I now understand well how nighttime media use can negatively affect sleep” and was committed to maintaining his current behavior. Information from the mother revealed that the patient was irritable at the beginning of the treatment, but not to the extent of being aggressive toward the family. She noted that the patient felt the effects of the treatment, becoming less sleepy and more active during the day. Additionally, the nighttime media use routine of the patient had shifted to the morning or early evening, leading to a reduced total screen time.

At the 16-week follow-up, both patient and mother recalled that treatment adherence and the effectiveness of the therapeutic intervention were maintained, with no adverse or unexpected events. Based on their subjective perspective, the key factors for staying motivated during treatment included increased family time, such as engaging in conversations with family members, and a clear understanding of the importance of avoiding nighttime media use at the onset of intervention. In the context of family involvement, receiving commendation from parents for adhering to night-time screen time restriction appears to have significantly motivated the patient.

## Discussion

Pediatric insomnia is recognized for its significance as an early sign or risk factor for mental illnesses and neurodevelopmental disorders. It affects up to a third of typically developing children and over 80% of children with neurodevelopmental disorders. The primary treatment options for pediatric insomnia are sleep hygiene education and behavioral therapy. However, owing to the lack of established evidence for generalized therapeutic interventions, it is crucial to consider the development of the child and involve parents in the treatment process (Meltzer et al., 2021). Additionally, it is important to comprehensively focus on outcomes beyond sleep variables over an extended period. Moreover, with the recent increase in screen time among children (Brzek et al., 2021), therapeutic interventions that consider the relationship between excessive screen time and pediatric sleep disturbance (Hisler et al., 2020) are being explored. However, to the best of our knowledge, there are no studies that have evaluated the effectiveness and safety of interventions aimed at forcibly reducing screen time during treatments of pediatric insomnia.

To summarize our research findings, in a case of chronic pediatric insomnia with typical development where the main causal factor is an increase in nighttime media use, enforced nighttime screen time restrictions following sleep hygiene education focused on screen time are effective in improving objective sleep variables over 2 weeks. Furthermore, the subjective therapeutic effects and adherence are maintained over 16 weeks without adverse events such as increased irritability or family conflicts.

In this context, this study is important for some reasons. The first key contribution of our study is the successful evaluation of increased TST in pediatric insomnia through enforced nighttime screen time restriction, objectively measured using actigraphy. In this case, reducing the nighttime screen time quickly increased the TST and decreased the ESS score. This finding contradicts the findings from previous RCTs (Das-Friebel et al., 2019; Knebel et al., 2020; Pedersen et al., 2022; Mahalingham et al., 2023), which did not identify any relationship between reduced screen time and an increased TST. Our study suggests the potential effectiveness of this approach for a subset of children with chronic insomnia where nighttime media use is a causal factor. Furthermore, employing an automatic time-limiting feature on digital devices may be a practical approach to decrease screen time. This aligns with reports that suggest significantly shorter screen time among users of this feature than among non-users (Arai et al., 2023), underscoring its potential efficacy as a therapeutic tool. The second key contribution is our discovery that enforced screen time restrictions following sleep hygiene education did not result in adverse events such as increased irritability or family conflict. To the best of our knowledge, there are no intervention studies that have evaluated the psychological safety of enforced screen time restrictions. Therefore, even with the limited condition of participants who are typically developing and have low SDQ scores, the assessment of safety in our study is important. Finally, we discovered that at the 16-week follow-up, both the patient and his mother identified increased family involvement as a crucial factor in maintaining adherence to the screen time restrictions. Consistent with this factor in maintaining adherence, Leonard and Khurana (2022) reported that a child's perception of their parents' involvement is inversely related to screen time before bedtime. Other than adherence maintenance factors that patients felt were effective, environmental factors, such as having a television in the bedroom (Falbe et al., 2015) and owning multiple devices (Ishtiaq et al., 2021), are reasons for increased screen time; this suggests that sleep clinicians should provide comprehensive sleep hygiene education related to media use.

It is important to consider the limitations of this study. The first limitation is that the characteristics of the patients (i.e., being 13 years old, typically developing, having low SDQ scores, with active family involvement during the period of treatment) limit the generalizability of our findings. The second limitation is that the use of subjective evaluations in assessing the long-term effectiveness of therapeutic interventions restricted our ability to determine the long-term effectiveness of the treatment. Considering these factors, future research is needed to investigate the effectiveness and safety of long-term enforced nighttime screen time restrictions using objective evaluations across a broader range of pediatric insomnia populations.

In conclusion, our study emphasizes the effectiveness of combining enforced nighttime screen time restrictions with sleep hygiene education to improve sleep variables in pediatric insomnia. This approach, when particularly applied in children with typical development and low SDQ scores, shows promise without causing adverse effects. However, the constrained scope that limits the generalization and the reliance on subjective evaluations underscore the importance of expanded research with more diverse populations and objective metrics to thoroughly ascertain the long-term outcomes and safety of this intervention.

## Data availability statement

The raw data supporting the conclusions of this article will be made available by the authors, without undue reservation.

## Ethics statement

The studies involving humans were approved by Ethics Committee of Kurita Hospital. The studies were conducted in accordance with the local legislation and institutional requirements. Written informed consent for participation in this study was provided by the participants' legal guardians/next of kin. Written informed consent was obtained from the individual(s), and minor(s)' legal guardian/next of kin, for the publication of any potentially identifiable images or data included in this article.

## Author contributions

YA: Conceptualization, Writing – original draft, Writing – review & editing, Data curation, Investigation. DS: Supervision, Writing – review & editing, Writing – original draft. KS: Supervision, Writing – review & editing. JW: Supervision, Writing – review & editing. YK: Supervision, Writing – review & editing. MK: Supervision, Writing – review & editing, Data curation, Investigation, Formal analysis. SW: Supervision, Writing – review & editing.
